# Supplementary material for: Mapping the evolution of fertility support policies in China: A content and instrumental analysis
Source: PLoS One. 2025 Oct 9;20(10):e0332137. doi: 10.1371/journal.pone.0332137 (PMC12510515; doi:10.1371/journal.pone.0332137)
Supplement: S1 Appendix — (ZIP) [file pone.0332137.s001.zip › S1 Appendix. 226 original policy documents/132-文化部关于印发《文化部机关国家公务员考勤办法》的通知(FBM-CLI-4-29743).doc]

文化部关于印发《文化部机关国家公务员考勤办法》的通知

**发布部门：** [文化部(已撤销)](https://www.pkulaw.com/law/chl?Aggs.IssueDepartment=60221) [机构沿革](javascript:void(0))

**发文字号：**文人发〔1995〕53号

**发布日期：**1995.11.21

**实施日期：**1995.11.21

**时效性：** [失效](https://www.pkulaw.com/law/chl?Aggs.TimelinessDic=02)

**效力级别：** [部门规章](https://www.pkulaw.com/law/chl?Aggs.EffectivenessDic=XE0301)

**法规类别：** [人事管理](https://www.pkulaw.com/law/chl?Aggs.Category=08804)

**失效依据：**本篇法规已被《文化部机关公务员休假暂行办法》（文人发〔2006〕34号）废止

　　　　　　　　　　　　　　 文化部关于印发
　　　　　　　　 《文化部机关国家公务员考勤办法》的通知
　　　　　（１９９５年１１月２１日　文人发（１９９５）５３号）

　　现将《文化部机关国家公务员考勤办法》印发给你们，请遵照执行。
　　　　　　　　　　　文化部机关国家公务员考勤办法

　第一章　总则

　　**第一条**　为加强文化部机关国家公务员的组织性、纪律性，规范各项请假、考勤制度，根据国家有关规定，结合部机关的实际情况，制定本办法。

　　**第二条**　本办法适用于文化部机关国家公务员。

　第二章　假期类别和期限

　　**第三条**　病假　公务员因疾病（因公致残或患职业病者除外）必须治疗和休养的，可以请病假。

　　**第四条**　事假　公务员因本人或家庭有紧急事务需要处理的，可以请事假。

　　**第五条**　公假　属下列情况之一者，经领导批准，可以算公假：
　　（一）公务员凭学校通知书参加学生家长会的。
　　（二）公务员的住宅设施因房管部门维修或其它原因须由公务员在家料理的。

　　**第六条**　婚假　公务员本人结婚，可以请婚假，假期三天。男二十五周岁、女二十三周岁以上初婚登记的，为晚婚。双方晚婚的，除国家规定的三天婚假外，各增加奖励假七天。

　　**第七条**　丧葬假　公务员的配偶、父母、子女、岳父母、公婆死亡，可以请丧葬假，假期不得超过五天。

　　**第八条**　探亲假　公务员连续工作满一年，与配偶或与父母不住在一起并不能在公休假日团聚的，可以请探亲假。
　　（一）公务员探望配偶的，每年给一方探亲假一次，假期三十天。
　　（二）未婚公务员（包括丧偶或离婚已满一年的）探望父母（包括自幼抚养其长大，现仍与本人保持联系的抚养人，下同），每年给假一次，假期二十天。如因工作需要，本司局当年不能给假或本人自愿两年探亲一次的，可以两年给假一次，假期四十五天。
　　（三）已婚公务员探望父母，每四年给假一次，假期二十天。

　　**第九条**　出境探亲假　凡符合国家规定探亲条件的公务员出境探亲，可以请出境探亲假，假期三个月，归侨、侨眷假期六个月。出境探亲的假期视不同情况可适当延长，假期最多不得超过一年。

　　**第十条**　路程假　公务员到外地结婚（不包括旅行结婚）、奔丧或探亲，给予往返路程假。

　　**第十一条**　生育假
　　（一）婚前检查假：公务员进行婚前检查，给假半天。
　　（二）产前检查假：女性公务员进行产前检查，按实际情况给假。
　　（三）产假：女性公务员产假九十天，其中产前假十五天；难产的增加假期十五天；多胞胎生育的，每多生育一个婴儿，增加产假十五天。
　　（四）晚育奖励假：已婚妇女二十四周岁以上生育第一个孩子的为晚育。晚育的女性公务员除国家规定的产假外，增加奖励假十五天。产妇护理有困难的，此项奖励假亦可由男方使用。
　　（五）抚育独生子女假：育龄女性公务员响应国家号召只生育一个孩子的，凭独生子女证，可以在产假期满后继续休假至孩子满六个月（晚育奖励假另加）。
　　（六）怀孕流产假：女性公务员怀孕流产的，根据医务部门的证明给予休假。
　　１．女性公务员怀孕不满四个月流产的，给假十五天至三十天。
　　２．女性公务员怀孕满四个月以上流产的，给假四十二天。
　　（七）哺乳假：女性公务员有不满一周岁的婴儿需要亲自哺乳的，每天给一小时的哺乳时间。多胞胎生育的，每多一个婴儿增加三十分钟。
　　以上（二）、（三）、（四）、（五）、（六）、（七）项均不适用非婚生育及非计划生育。

　　**第十二条**　工伤假　公务员因公受伤，必须治疗和休养的，给予公伤假。

　　**第十三条**　搬迁假　公务员因住房搬迁，给假三天。

　　**第十四条**　出差假　公务员因公出差前，根据实际情况可做有关准备事宜，给假半天至一天；完成任务返回后，出差超过两周的，给假一天，超过一个月的，给假两天。对于出差的公务员的公休假日，应在出差的地点享受，如果在出差地点未享受的，应在两周内补给与公休假日相等的休息时间。

　　**第十五条**　值班假　公务员在法定假日或公休假日值班的，应在两周内补给与值班时间相等的休息时间。

　　**第十六条**　出国准备假　派驻使领馆常驻人员出国前，给假三十天，准备出国事宜。

　　**第十七条**　驻外回国休假　派驻使领馆常驻人员，任期结束回国后，给假三十天，未在任期内休假的，给假六十天，中间休假超假的不再给假。

　　**第十八条**　带薪年休假　公务员（不包括试用期和见习期人员）连续工作一年以上，可享受带薪年休假。
　　（一）参加工作不足十年的，休假七天；
　　（二）参加工作满十年不足二十年的，休假十天；
　　（三）参加工作满二十年的，休假十五天。

　　**第十九条**　假期计算　病假、生育假、路程假、探亲假、出境探亲假、公伤假、出差假、出国准备假、驻外回国休假均包括法定假日和公休假日在内；事假、婚假、丧葬假、值班假、带薪年休假均不包括法定假日和公休假日在内。

　第三章　各类假期的待遇

　　**第二十条**　病假期间的待遇　全年病假累计超过三个月不满六个月的，扣发本人年度奖金的三分之一；超过六个月的，不发本人年度奖金。

　　**第二十一条**　事假期间的待遇　全年事假累计超过一个月不满三个月的，扣发本人年度奖金的三分之一；超过三个月不满六个月的，扣发本人年度奖金的二分之一；超过六个月的，不发本人年度奖金。

　　**第二十二条**　探亲假期间的待遇　探亲假期间，年度奖金照发。

　　**第二十三条**　出境探亲假期间的待遇　出境探亲假期间，年度奖金照发。批准续假期间内待遇，按事假规定办理。

　　**第二十四条**　生育假期间的待遇
　　（一）生育假期间，年度奖金照发。
　　（二）产假期满后，因身体原因仍不能工作的，经医务部门证明后，其超过产假期间的待遇，按病假的有关规定处理。
　　以上（一）、（二）项均不适用于非婚生育及非计划生育。

　　**第二十五条**　各类假期间的工资待遇执行有关规定。

　第四章　请假程序

　　**第二十六条**　公务员请假须提出书面申请，填写《文化部机关国家公务员请假单》（附件一）①，经领导批准后有效。如因重病或急事不能事先请假时，可委托他人代为请假或办理补假手续。①　附件一略。

　　**第二十七条**　凡请病假或工伤假两天以上的，均须有合同医院证明或非合同医院的急诊证明；病假、工伤假需延长休养期和产假期满需继续休养的，均根据合同医院的诊断证明由所在司局审核批准。

　　**第二十八条**　请假期满因故不能上班的，可以申请续假。续假手续与请假手续相同。
　　公务员出境探亲一般不得续假，如确有特殊情况，不能按期返回的，本人必须提前一个月向所在司局提出申请，经批准方可续假。

　　**第二十九条**　请假或续假期满上班后，应立即销假。

　　**第三十条**　公务员请假，假期在一周之内的由直接行政首长审批，超过一周的应由上一级行政首长审批。司局长请假，由主管部长批准并报办公厅部长办公室备案。司局长的考勤由各司局办公室负责。

　第五章　旷工及其行政处理

　　**第三十一条**　凡属下列情况之一者，按旷工论：
　　（一）不经请假或请假未获批准而擅自离开工作岗位的。
　　（二）请假期限已满，不续假或续假未获批准而逾期不归的。
　　（三）不服从组织调动和工作分配，不按时到工作岗位工作的。

　　**第三十二条**　旷工连续十五天以内或一年累计三十天以内的，不发年度奖金。

　　**第三十三条**　旷工连续超过十五天或一年内累计超过三十天的，予以辞退。

　第六章　附则

　　**第三十四条**　本办法的实施由各司局办公室（综合处）负责。各司局应建立相应的考勤制度，每月由各处（室）填写《文化部机关国家公务员考勤登记表》（附件二）①，定期公布公务员出勤、迟到、早退、请假、旷工等情况，各司局办公室（综合处）汇总后，于每月十日前，将《考勤登记表》报送人事司，其中涉及工资变动、年度奖金和行政处理的，由人事部门承办。①　附件二略

　　**第三十五条**　本办法由人事司负责解释。

　　**第三十六条**　本办法自公布之日起施行。

© 北大法宝：（[www.pkulaw.com](https://www.pkulaw.com/)) 专业提供法律信息、法学知识和法律软件领域各类解决方案。北大法宝为您提供丰富的参考资料，正式引用法规条文时请与标准文本核对。
欢迎查看所有[产品和服务](https://www.pkulaw.com/helps/2.html)。法宝快讯：[如何快速找到您需要的检索结果？](https://www.pkulaw.com/helps/70.html)[法宝](https://www.pkulaw.com/helps/69.html)V6有何新特色？

扫描二维码阅读原文

**原文链接：**<https://www.pkulaw.com/chl/09c757b94ff5091abdfb.html>
